# The Inherent Statistical Difference of Particular Hospitals

**Published:** 1916-03-04

**Authors:** 


### KING EDWARD'S HOSPITAL FUND.

### The Inherent Statistical Difference of Particular Hospitals.

THE King's Fund last return of the figures of the hospitals of London deals with 108 institutions. These consist of:

Eleven general hospitals with medical schools, which had an average of over one hundred beds in daily occupation during 1914.

Eight general hospitals without medical schools, which had an average of over one hundred beds in daily occupation during 1914.

Fourteen general hospitals, which had an average of under one hundred beds in daily occupation during 1914.

Three consumption hospitals, six hospitals for women, six hospitals for children, five ophthalmic hospitals, three hospitals for epilepsy and paralysis, thirteen cottage hospitals, eight lying-in hospitals, thirty-one unclassified hospitals.

Full in-patient and out-patient statistics of all these hospitals are given, and the total ordinary expenditure is shown, tabulated under the regular sub-headings belonging to the maintenance and administrative sections of the income and expenditure account. Further information is afforded, or rather the information is tabulated in a different manner, in reference to seventy-seven of the hospitals—*i.e.*, those which are not described as "unclassified hospitals"; these additional tables are designated "group comparisons." Previous to 1914 the institutions contained in the first and second groups in the list given above were compared together, but by general desire the hospitals without medical schools are now separated for purposes of group comparison from the larger non-teaching hospitals.

Apart from the question of teaching, the principle that size is an important factor in separating our hospitals for statistical study seems to have been accepted for the purpose of grouping general hospitals. In respect, however, of institutions devoted to children, to women's diseases, or to special classes of disease, size is not considered, the one guide for classification being the nature of the hospital.

The result is that we have before us four pages of tables in respect of each of seven groups, an arrangement which, with the exception of one or two cases, is not very helpful to the student of the statistics as a whole, as the result it produces is merely the average cost per occupied bed or per out-patient attendance of a group of institutions which are similar only in respect of the nature of the diseases treated, or the sex or age of the patients.

For reasons that are apparent to most observers of hospital affairs, there is as little to be gained by comparing the general statistics of, say, the Seamen's (*Dreadnought*) Hospital with those of the King Edward Memorial Hospital at Ealing, as by considering side by side the records of Brompton Consumption Hospital and the Royal Hospital for Diseases of the Chest, the Hospital for Sick Children and the Belgrave, the Royal London Ophthalmic and its lesser sisters, or the National Hospital for the Paralysed and the West End Hospital for Nervous Diseases. One might as well compare a *Dreadnought* with a mine-sweeper, a grand piano with a cottage piano, or a thousand-pound automobile with a motor-bicycle. Each has its uses, and the work of the smaller type is not less admirable because it is not designed for larger effect or activities. What is the inherent difference for purposes of statistical comparison between a general hospital and a special hospital or between a hospital for women and a hospital for consumption which should have an effective influence in the arrangement of these tables, entailing, in these times when convenience in the form of presentation of information is such a boon, the issue of a book of eighty-four pages where one of sixty or less would have answered its purpose as well, if not better?

Does the difference lie in the treatment or feeding of the patient, the size of the staff, or the equipment of the institution? To examine the question from every point of view let us compare general hospitals with special hospitals, and special hospitals of one kind with those of another, under the headings which should express in money figures all the considerations we have quoted, taking size as the chief principle under which our group table is drawn up. As 1914 was, by reason of the special effect on expenditure caused by the war, an abnormal year, the figures for 1913 are for this purpose employed.

It would help us enormously in our examination of these comparative figures if we were in possession of another item of information—namely, the number of "mouths to feed" at each institution. In the last article on this subject it was pointed out that proper investigation is baffled at almost every point because of the absence of this essential information. It would be quite easy to provide in the returns a column for the "daily average number of patients and employees resident," and as it is promised that suggestions of alterations in the methods of presenting the statistics will receive the most careful consideration of King Edward's Hospital Fund, we venture to ask for this simple addition to the valuable returns, leaving any question of working out the average cost per occupant of the hospital (patients plus staff) for future consideration.

We have above details of four general hospitals, a children's hospital, and three special hospitals, the latter treating diseases which in each case require equipment and general arrangements very different in many respects from those at most other institutions. Yet in the whole table the divergence between the highest and lowest cost per occupied bed, either as regards the total cost or that under headings, is not wider than that shown in most of the group tables in the statistical return. But there are points, when we come to examine the figures in detail, upon which a case can be argued for the present group arrangement in some form. It is clear, for instance, that the general hospitals, with a large proportion of surgical beds and consequent heavy expenditure under "Surgery and Dispensary," can with advantage to the student of the figures be grouped as at present. The hospitals for children, too, by reason of the age of the majority of the inmates, always show a low cost under "Provisions," and there are a sufficient number, the majority not differing very widely in point of size, to make an interesting group table. The total beds occupied on the average every day at the six women's hospitals range from 17 to 67 (four of them from 42 to 67). Here, again, is material for an interesting group comparison. But we should like to see the saving of a great deal of space, and the provision of information in a much more convenient form, by the grouping of the special hospitals in two or three lists according to size. From the instances given in our table it is gathered that the peculiarities of the institutions have not resulted in any striking differences as to cost; it seems a pity to separate them, especially as by so doing the reduced data provided is inadequate as the basis of conclusions as to economy. If in addition the number of "mouths to feed" is stated, common knowledge as to the requirements of each branch of hospital work will be the chief thing required of the reader of the tables. The editor of the statistical return will then be able to bring into his group comparisons for the first time a number of institutions, the work of which, as expressed by figures, will have enhanced interest when examined side by side with the information of other hospitals of a special character and similar size.

It will be noticed that we have not touched at all upon the out-patient side of this question. It is true that specialism causes a great variation as between institutions in respect of the cost per out-patient or per out-patient attendance. But, after all, the great bulk of the income of a hospital is spent upon its in-patient department, and as a rough gauge of economy the public always makes use of the cost per occupied bed.

General and Special Hospitals of from 150 to 200 Beds.

| Name | Beds Occupied | Cost per Occupied Bed under Headings: Provisions | Surgery and Dispensary | Domestic | Salaries and Wages | Management (other than Official Salaries and Pensions) | Total | Out-Patients' Cost per Attendance under Same Headings |
|---|---|---|---|---|---|---|---|---|
| | | £ s. d. | £ s. d. | £ s. d. | £ s. d. | £ s. d. | £ s. d. | Pence |
| Great Northern Central (General) ... | 176 | 24 9 9 | 10 4 11 | 19 8 5 | 25 2 4 | 1 4 8 | 80 10 1 | 7·68 |
| Westminster (General) ... ... ... | 183 | 29 1 1 | 11 17 5 | 20 7 10 | 28 0 7 | 0 10 10 | 89 17 9 | 9·47 |
| City of London (Chest) ... ... ... | 163 | 24 18 4 | 2 4 3 | 13 17 2 | 18 13 8 | 1 7 5 | 61 0 10 | 12·0 |
| National Paralysed ... ... ... | 153 | 25 2 9 | 4 0 6 | 18 12 2 | 26 8 3 | 1 1 4 | 75 5 0 | 9·74 |
| **General and Special Hospitals of from 100 to 150 Beds.** | | | | | | | | |
| Charing Cross (General) ... ... ... | 141 | 26 2 10 | 7 2 7 | 24 18 11 | 30 13 2 | 0 14 2 | 89 11 8 | 8·81 |
| German (General) ... ... ... | 128 | 31 10 10 | 12 5 11 | 15 13 3 | 16 4 7 | 1 5 3 | 76 19 10 | 6·46 |
| East London (Children) ... ... ... | 113 | 16 4 7 | 6 9 1 | 17 4 9 | 24 2 2 | 1 3 10 | 65 4 5 | 6·30 |
| Royal London Ophthalmic ... ... | 111 | 20 4 5 | 3 7 7 | 14 5 5 | 19 0 6 | 0 7 6 | 57 5 5 | 5·51 |

Note.—Previous articles appeared on Nov. 6, p. 125; Nov. 27, p. 185; and Dec. 11, 1915, p. 227.

